# Children’s Sex and the Happiness of Parents

**DOI:** 10.1007/s10680-016-9387-z

**Published:** 2016-08-22

**Authors:** Rachel Margolis, Mikko Myrskyla

**Affiliations:** 1 Department of Sociology, Room 5326, Social Science Centre, The University of Western Ontario, London, ON N6A 5C2 Canada; 2 Max Planck Institute for Demographic Research, Konrad-Zuse-Str 1, 18057 Rostock, Germany; 3 Department of Social Policy, London School of Economics, London, UK; 4 Population Research Unit, University of Helsinki, Helsinki, Finland

**Keywords:** Parity progression, Sex preferences, Low fertility, Happiness

## Abstract

Demographers are interested in sex preferences for children because they can skew sex ratios and influence population-level fertility, parenting behavior, and family outcomes. Based on parity progression ratios, in most European countries, there are no sex preferences for a first child, but a strong preference for mixed-sex children. We hypothesize that mixed-sex preferences also influence parental happiness. Parents’ disappointment with a second child of the same sex as the first could have negative effects for parents and children. We use longitudinal data from the German Socio-Economic Panel and the British Household Panel Study to examine parental happiness by the children’s sex and analyze whether these effects differ by parent’s sex, age, nativity, and educational attainment. The results are only partially consistent with predictions from parity progression ratios. As expected, parental happiness does not depend on the sex of the first child. We find weak evidence suggesting that two boys decrease happiness, but the findings are not consistent across German and British data or across subpopulations. Moreover, two girls do not reduce happiness. Although sex preferences influence fertility, they appear to have little impact on happiness, perhaps because of unobserved positive factors associated with having same-sex children.

## Introduction

Demographers have long been concerned with parents’ sex preferences of their children. Sex preferences can substantially increase the level of fertility if couples continue to have children until they have the number of desired sons, daughters, or mix that they desire (Bongaarts and Potter 1983). More broadly, sex preferences are a public issue because son preference combined with sex-selection technologies has led to skewed sex ratios in many parts of Asia (Park and Cho 1995; Li et al. 2000; Johansson and Nygren 1991) and recently similar skews were documented among immigrant populations in Canada (Almond et al. 2013). Although there has been much less research on this topic in European countries, sex preferences still exist (Andersson et al. 2006; Brockmann 2001; Hank and Kohler 2000) and can be a very important factor affecting the level of fertility in low fertility settings (Wood and Bean 1977; Bongaarts 2001). In fact, many studies have found a consistent preference for having at least one child of each sex in many European countries. These sex preferences are documented from either parity progression ratios, that is, the proportion going on to have an additional child based on the sex composition of existing children, or intentions to have another child based on a given sex composition (Andersson et al. 2006; Brockmann 2001; Hank and Kohler 2000).

In traditional contexts with strong son preferences, daughters often receive differential treatment in terms of nutritional and health resources within the household (Lundberg 2005; Strauss and Thomas 1995; Thomas 1994). There is little evidence of this type of sex-based discrimination based on resources in Europe and North America, and this is thought to be because public and private pension provision has eroded son preference, parents can afford to treat all of their children well, and the gender revolution led to cultural change in childrearing practices. In fact, Taubman (1991) finds few differences in the treatment of boy and girl children in terms of bequests, transfers, and education. Given the seemingly equal treatment of boys and girls, it is notable that there are differences in some aspects of parenting behavior and family outcomes based on the sex of children (Lundberg 2005). For example, in the USA, fathers spend more time with sons than daughters, and mothers with sons report greater marital happiness (Raley and Bianchi 2006). However, no research has investigated whether the preference for mixed-sex children in Europe translates into differential parental happiness.

In this paper, we examine whether we can learn about sex preferences from parents’ happiness after a child is born, and whether parent’s happiness maps onto parents’ behavior measured with parity progression. Prior research on European countries suggests that there is no preference for boys or girls, except after the birth of the first child when the preference becomes to have a child of different sex. We therefore hypothesize that the sex of the first child does not influence parental well-being, but if the first two children are the same sex, parental well-being should be dropped. We test our prediction using nationally representative longitudinal data sets from Germany and Britain.

The topic is important because if parents are disappointed by the sex composition of their children—which parity progression ratios suggest is happening if the second child is same sex as the first—the parents’ subjective well-being and mental health could suffer and translate into negative effects on children. Prior research on fertility and well-being of the parents has focused on the association between the number of children, timing of children, and whether the associations between birth of a child and changes in well-being are permanent or transitory. The evidence is mixed. According to a review by McLanahan and Adams (1987), no study (at that time) had found that parents would be better off by any conventional measure of well-being than childless people. A more recent review by Hansen (2012) also states that most of the evidence suggests that people are better off without having children. Research focusing directly on subjective well-being, or happiness, however, is not that bleak about the potential impact of children on happiness. Kohler et al. (2005) analyze Danish twins and document a strong increase in happiness among those who have one child. Clark et al. (2008) conduct a longitudinal analysis that combines all parities, and find that while the birth of a child increases happiness, the impact is short-lived. Margolis and Myrskylä (2011) and Aassve et al. (2012) both show that the association between the number of children and happiness varies across countries and is sensitive to the context.

For our analysis, a particularly important study is Myrskylä and Margolis (2014), as their analysis covers the same two countries as the current paper, Germany and United Kingdom, and the findings provide important motivation for our analysis. Myrskylä and Margolis (2014) analyze the longitudinal association between happiness and fertility and report the same global finding that Clark et al. (2008) delivered: children increase happiness only temporarily. However, the association varies by parity so that although the increase in happiness associated with first birth is large, the increase associated with the second birth is only about half of the first. It is possible that the increase in happiness associated with a first birth is particularly large because assuming no gender preferences, there can be no disappointment. With the second child, however, there is close to 50 % chance of disappointment if the parents desire one of both sexes. Margolis and Myrskylä (2015) also show that parental happiness is an important predictor of further parity progression, suggesting that the links between fertility and happiness are both short and long term and have consequences for further fertility behavior.

However, it is also possible that even strong sex preferences do not translate into differences in parental happiness due to countervailing forces. Consider the explicit prediction of this paper that parents with two same-sex children are less happy than parents with one of each. Two same-sex children may also positively influence parental happiness. Potential mechanisms could include mundane issues such as increased possibilities to recycle clothes and other items from the older sibling to the younger. If two same-sex children elicit the desire to have an additional child, this may also influence happiness by binding the parents together through an unfinished agenda. Finally, whatever disappointment there may be with the sex of the second child, this effect may be short-lived, as are the effects of many important life events (Clark et al. 2008).

### Sex Preferences in Europe

Given people’s decreasing claim to have sex preferences over time, demographers infer sex preferences from behavior. Parity progression analyses examine the proportion of parents to have another child given the number and sex combination of existing children. By measuring actual behavior, we can infer sex preferences. The second and less common way in which sex preferences are documented is by examining fertility intentions given the sex of existing children. Using survey data, one can estimate the likelihood of intending to have an additional child(children) given the set of children that one already has.

Compared to research on children’s sex preferences in Asia, Africa, and the Americas, there is much less research on sex preferences in Europe. There are two main findings from the European context. First, there are few sex preferences affecting progression to a second birth. For a first child, there is no effect of sex on parity progression in Denmark, Finland, Norway, and Sweden (Andersson et al. 2006). There is a slight preference for daughters in Portugal and in eastern Germany (Brockmann 2001; Hank and Kohler 2000) and a slight preference for boys in western Germany (Hank and Kohler 2000) although the West German finding was not found in Brockmann’s study (2001). Second, there is a consistent desire to have mixed-sex families (Hank and Kohler 2000). This is found in Austria, Belgium, Czech Republic, East Germany, Hungary, Italy, Latvia, Lithuania, Slovenia, Spain, Sweden, Denmark, and Switzerland, but not in Finland, France, West Germany, Norway, Poland, and Portugal (Andersson et al. 2006; Hank and Kohler 2000) and the UK (Dahl et al. 2006).

### Consequences of Sex Preferences

At the macro level, sex preferences can have large effects on the population level of fertility, especially in low fertility settings. Strong desires for a particular sex composition lead to substantially higher fertility. For example, if couples have children until they have at least one son, TFR will be 1.94. If they continue until they have at least one daughter, it will be 2.06. If they want one daughter and one son, then they will have on average three children (Bongaarts and Potter 1983).

The micro-level effects of sex preferences have gotten less attention but are not unimportant. Fathers’ investments in children tend to be somewhat higher in families with sons, they spend more time with sons than daughters, and more often stay in a marriage if there are sons (Raley and Bianchi 2006). There are two reasons for these patterns. First, parent–child relationships may be symmetric or asymmetric based on the gender of parents and children, especially if there is gender differentiation within the family (Williamson 1976). While the differences in labor market outcomes by sex in Europe have been declining, there continue to be important differences in work hours (Antecol 2000; Sayer 2005) and also in how much family and care work adult children do for their parents (Bolin et al. 2008; Haberkern and Szydlik 2010). Therefore, parents might want one daughter and one son in order to ensure a child filling each of these roles. Another reason is that parents might derive different pleasure from seeing sons and daughters grow up and doing certain activities with them (Bulatao 1981; Friedman et al. 1994; Hoffman and Manis 1979). For example, some activities may be enjoyed more by sons or daughters such as sports, outdoor activities, or shopping. Parents’ happiness could differ if they get to do more or less of these things that they value, based on sex combinations of the children. In addition, parents may value variation.

### The Current Study

In this paper, we examine three research questions. First, we examine whether the sex of the first child or sex of the first two children affects parity progression in the UK and Germany. We replicate the findings from prior research on this topic. Second, we examine whether the sex of the first and first two children affects parents’ happiness. Our first hypothesis is that parents’ happiness will vary based on children’s sex. These differences will mirror the preferences for mixed-sex families that we observe in parity progression. An alternative hypothesis is that there will be no effect of children’s sex on parents’ happiness. Perhaps, there will be a short-term effect when finding out the child’s sex, but that parents will get over this short-term disappointment and we will see few strong medium-term effects on happiness. We test whether sex preferences exist in each year until children are ten years old.

Third, we examine whether the effects of children’s sex on parental happiness differ by the parent’s sex, education level, nativity, and age. Parents may derive more enjoyment from doing activities with children of the same sex. Therefore, we test whether men or women have different happiness responses to different sex combinations of children. Sex preferences may be more or less distinct by the education level of parents. Those with less education and more entrenched traditional gender roles may have stronger sex preferences for children than those with less traditional gender norms. The effect of children’s sex on the happiness of parents also might vary by nativity. We would expect that immigrants to Europe would have stronger son preferences if they are coming from societies where these beliefs are widespread. Finally, we analyze the sex preferences revealed by parental happiness by the age at which one becomes parent or has the second child. Assuming parents desire one of each, the second child being same sex as the first may be a particularly large disappointment for older parents who may have less time and opportunities to have a third child.

## Data

We use the German Socio-Economic Panel (SOEP) and the British Household Panel Survey (BHPS). The SOEP is a representative longitudinal study of households including Germans living in the old (West) and new (East) German states, foreigners, and recent immigrants to Germany (Wagner et al. 2007). The SOEP started in 1984 with the new German states added in 1991. The BHPS is an annual survey that started in 1991, consisting of a nationally representative sample of households. We chose these two countries as the context for the study because these are two of the largest countries in Europe; together, they cover both a broad range of fertility regimes from lowest-low fertility (Germany having had below 1.3 TFR in the early 1990s) and moderately high modern European fertility (UK having had TFR between 1.6 and 1.9 since the 1991); they both have large immigrant populations to test whether patterns vary by nativity; and they have high-quality data with which to test our hypotheses. Both of these data sets are very large, representative surveys with many measures of happiness, life satisfaction, and the number and sex composition of children. The surveys also have long enough follow-up to examine short- and medium-term effects of children’s sex on parental happiness and parity progression.

Our analysis draws on survey waves from 1984 to 2013 in Germany and 1991–2012 in the United Kingdom. For the parity progression analysis, we include individuals whose first or second birth was observed within the time window. In the SOEP, there were 6001 observed first births and 2988 observed second births. In the BHPS, the corresponding numbers are 7012 first births and 3113 second births. These are the respondents for whom children’s sex might have some effect on happiness or future reproductive behavior. After exclusion of those who had twins, triplets, or missing data, the resulting sample sizes are 4810 respondents (SOEP) and 5042 (BHPS) for progression to parity two, and 2482 (SOEP) and 2013 (BHPS) for progression to parity three.

Sample sizes of the happiness regressions are larger than in the parity progression analysis because we are able to include persons who had children before they enrolled in the sample. For example, if someone had a child × years before entering the survey, we observe this person’s happiness × years after the birth and include in the analysis as long as *x* is less than 10 years. The sample sizes for the happiness regressions are therefore 5532 and 5299 persons for first birth analysis for SOEP and BHPS, respectively, and 4115 and 4247 persons for second birth analysis for SOEP and BHPS, respectively.

To answer the question of how sex of the child or children influences happiness, we analyze happiness from one year before the birth of the first or second child (to capture effects during pregnancy, when the sex of the child may already be known) until 10 years after the birth. With an increasing lag, the sample sizes get smaller. To increase statistical power, we include also parents whose children were born before the observation period.

**Fig. 1:**
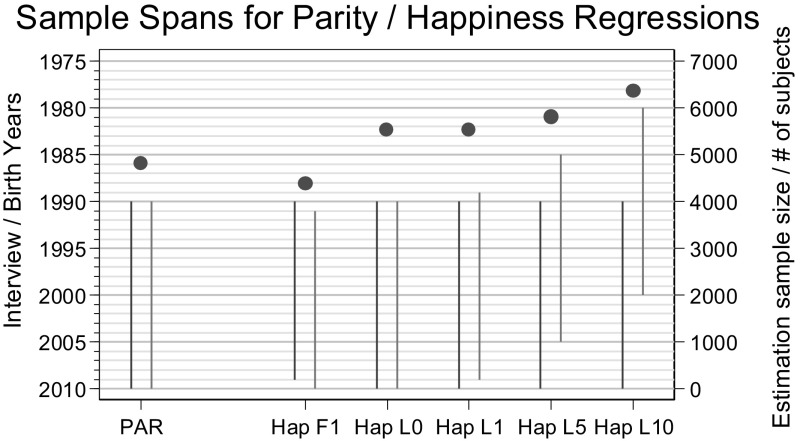
Graph showing how the sample evolves with lags. *Note* *Blue lines* represent parents (interview years) considered, *red lines* years of births considered (l*eft-hand scale*). A sample is determined by a combination of the two lines. Read the years 1990 and 2010 as ‘survey start’ and ‘survey end’; exact dates are 1991–2012 (BHPS) and 1984–2013 (SOEP). *Dots* are SOEP sample sizes for first-order births (*right-hand scale*). (Color figure online)

Figure 1 illustrates the sample selection process for both the parity progression analysis and happiness analyses. The samples are approximately equal in the analysis of parity progression and happiness in the year when the child was born. For the analysis of happiness in the year before the child is born, the sample is slightly smaller. In the analysis of long-term effects of child sex on happiness, the longer the lag gets, the more recent the parents are excluded and the parents who had children in the years preceding the start of the survey are included.

### Measures

We analyze two dependent variables: parity progression and subjective well-being of parents. Parity progression is derived from births of children, which are indicated by a change in the number of biological children reported in the birth biography data. The birth biography data also include the sex of the child. We exclude stepchildren or adopted children from the analysis because they are not observed in the data.

We use two slightly different questions to measure parental well-being in the German and British data. In the German sample, our measure is based on the question, “How satisfied are you with your life, all things considered?” with responses range from zero (completely dissatisfied) to ten (completely satisfied). In the British sample, parental well-being is measured with the question, “Have you recently been feeling reasonably happy, all things considered?” with responses ranging from one (much less happy than usual) to four (more happy than usual). The BHPS also includes another question on happiness: “How dissatisfied or satisfied are you with your life overall,” with answers ranging from one (not satisfied at all) to seven (completely satisfied). The latter life satisfaction however is not asked consistently through waves, so we focus on the general happiness question which measured consistently through all the BHPS waves. Although questions about happiness and life satisfaction are not exactly the same, they both capture positive aspects of subjective well-being. Moreover, prior research suggests that associations between childbearing and these two subjective well-being variables are highly similar (Myrskylä and Margolis 2014). We rescale the 4-point general happiness variable used in the BHPS that has values 1, 2, 3, and 4 to range from zero to ten (by subtracting 1 and multiplying by 10/3) to allow comparison of the magnitude of the coefficients across BHPS and SOEP.

Other variables used are the sex of the parent, age at birth of the first or second child, education at the time of the birth of the first or second child, and country of origin. In the German data, education is measured in years. In the British data, education is measured with six categories. Different variables of the SOEP and BHPS are mapped into a dichotomous high/low education level. For the BHPS, categories “college of further education,” “polytechnic,” and “university” are classified as “high education level”; categories “nursing school,” “other training establishment,” and none of the above” are classified as “low education level”. Country of origin is classified in both countries into native born versus non-native born; in the SOEP data, natives include those who migrated before the year 1949. The sample sizes are not large enough to distinguish between different countries of origin.

## Method

Our methodological approach rests on the assumption that child sex is exogenously determined. While recent research has shown that in Western countries, there may be subpopulations with skewed sex ratios at birth (Almond et al. 2013); suggesting sex-selective abortion, these deviations apply to small subpopulations. At the total population level, we argue that the assumption of exogenously determined distribution of children’s sex, while perhaps not strictly true, is still close to reality. Under this assumption, we do not need to worry much unobserved selection or control variables. Therefore, our models are simple.

In the analysis of parity progression, we use Cox proportional hazard models to estimate the relative risk of having an additional child depending on the sex distribution of existing children. We estimate separate models for progression to parity two and progression to parity three. In progression to parity two, the key predictor is an indicator for whether the first child was a boy. As control variable, only age at first birth is included. In progression to parity three, we again control only for age at second birth, and the key predictors are whether the first two children were both boys, both girls, or one of each. In both regressions, individuals are censored at the end of the survey or at age 45, whichever happens first. To analyze whether the impact of child sex on further parity progression depends on individual characteristics, we also estimate models that interact child sex with the sex of the parent; education (high vs. low); age of the parent (<25, 25–34, 35+); and country of origin.

The analysis of the effect of children’s sex on happiness is based on a series of regressions in which a separate model is estimated for happiness before and after the birth of the child. To capture the potential effect on happiness during pregnancy, we estimate a model in which happiness in the interview preceding the birth (year “−1”) of the child is regressed on the sex of the child. A separate model is estimated for first births using indicator for whether the child was boy as a predictor, and second births using indicators for whether the children were both boys or both girls as predictors. Next, we estimate similar models regressing happiness on the sex distribution of children but now analyzing happiness in the interview after the birth of the child (year “0”). We continue estimating such regressions until the 11th interview after the birth of the first or second child (year “10”). We then graph the point estimates of happiness over the years from −1 to +10 to analyze how, if at all, happiness changes in response to the sex of the children. As in the parity progression analysis, we control only for age at birth; in addition, we estimate interaction models by sex of the parent, age, education, and country of origin. We use linear regressions since others have found that treating life satisfaction as ordinal versus cardinal makes little difference (Ferrer-i-Carbonell and Frijters 2004).

In the analysis of the impact of same-sex versus mixed-sex distribution of children on happiness, we keep the parents who have a third birth in the analysis also after the birth of the third child. This is important as otherwise the group that has two children would, over time, start to converge to a group that is just content with the two children they have as those who are not content to move to parity three, and this would potentially bias our estimates of the effect of the sex distribution of children on happiness.

## Results

First, we examine sample characteristics for the BHPS and SOEP. Table 1 presents descriptive statistics including the sample sizes, demographic characteristics, and information about the happiness of respondents and children’s sex. The sample sizes for the German and British data at the time of the first child are both larger than five thousand and are smaller for the analysis of the second child. Just more than half of respondents have a boy for their first child, corresponding to normal sex ratios at birth that are slightly above 1.0. Among those that have two children, approximately a quarter have two boys, another quarter two girls, and half have one of each. Average levels of happiness are high, mostly around 7 on a 0–10 scale. Most births occur to respondents between the ages of 25 and 34, with smaller proportions to older and younger respondents. Between two-third and three-quarter of respondents go on to have a second child within ten years, and between 30 and 40 % of those having a second child go on to have a third. About four in ten Germans and two in ten of those in the UK have a high level of education at the time of a birth. Last, the majority of German respondents are native born, while about half of respondents in the UK are immigrants.

**Table 1:** Sample characteristics

	SOEP		BHPS	
	First child	Second child	First child	Second child
Number of respondents	5532	4115	5299	4247
% Female (of respondents)	57.5	56.7	57.0	58.3
Child sex (%)				
First child boy	50.1	–	51.1	–
First child girl	49.9	–	48.9	–
First two: one boy and one girl	–	49.2	–	49.7
First two boys	–	26.4	–	26.4
First two girls	–	24.4	–	23.9
Happiness, mean^a^				
At birth	7.6	7.4	7.1	6.8
After 10 years	7.0	7.0	6.5	6.5
Age at birth (%)				
<25	21.1	7.7	26.1	11.8
25–34	61.8	62.3	54.8	56.4
35+	17.1	30	19.2	31.7
% Having an additional birth within 10 years^b^	68.5	30.8	73.4	39.9
% High education at the time of birth^c^	42.5	41.4	20.7	21.3
% Born in Germany/United Kingdom	81.9	76.3	47.2	49.6

*SOEP* German Socio-Economic Panel 1984–2013, *BHPS* British Household Panel Survey 1991–2012

^a^Happiness is measured as the “Overall life satisfaction” on an scale 0–10 (SOEP) and as “General Happiness” on a scale 1–4 (BHPS). The BHPS happiness numbers have been rescaled to match the scale of the SOEP

^b^Cases of multiple births per year, including the births of twins and triples, have been excluded from the sample

^c^Education level is recorded as of birth of the first (second) child. Different variables of the SOEP and BHPS are mapped into a dichotomous high/low education level. For the BHPS, categories “nursing school, etc.,” “other training establishment,” and “none of the above” are classified as “low education level”; categories “college of further education,” “polytechnic,” and “university” are classified as “high education level”. For the SOEP, a high education level is defined as 12 or more years of education. “Born in Germany” includes immigrants before 1949

Next, we examine whether the sex of the first child, and sex of the first two children predicts progression to the next higher order birth. Table 2 presents results from Cox proportional hazard models predicting progression to a second and third birth, based on the sex of the existing children. The first two columns show the hazard of having a second birth based on the sex of a first child. We find that in both the UK and Germany that there is no difference in the likelihood of having a second child based on the sex of child one. The hazard ratios are 1.03 and 1.04 and are not significantly different from 1.0.

**Table 2:** Hazard of the next birth from Cox proportional hazard models, exponentiated coefficients

	Progression to second birth		Progression to third birth	
	BHPS	SOEP	BHPS	SOEP
Sex of first child				
First child is boy (girl)	1.03	1.04		
Sex of first and second children				
First two are boys (one boy and one girl)			1.34**	1.39***
First two are girls (one boy and one girl)			1.55***	1.36***
Age of parent	1.00	0.98***	0.92***	0.89***
No. of respondents	5042	4810	2013	2482
No. of births	2314	2664	428	626

* *p* < 0.10; ** *p* < 0.05; *** *p* < 0.01

We further examine whether there are differences in parity progression to a third birth, given having two boys or two girls, relative to having one child of each sex. In both countries, we find strong effects of sex composition on progression to a third birth. In the UK, there is a 34 % higher hazard of a third birth given the first two being boys and a 55 % higher hazard given two girls. In Germany, the respective hazard ratios are 39 and 36 % higher for two boys and two girls than having mixed-sex children. In sum, there do not seem to be sex preferences for progression to a second child, but these are strong ones for mixed sex which are found in a higher likelihood of progression to a third birth.

**Fig. 2:**
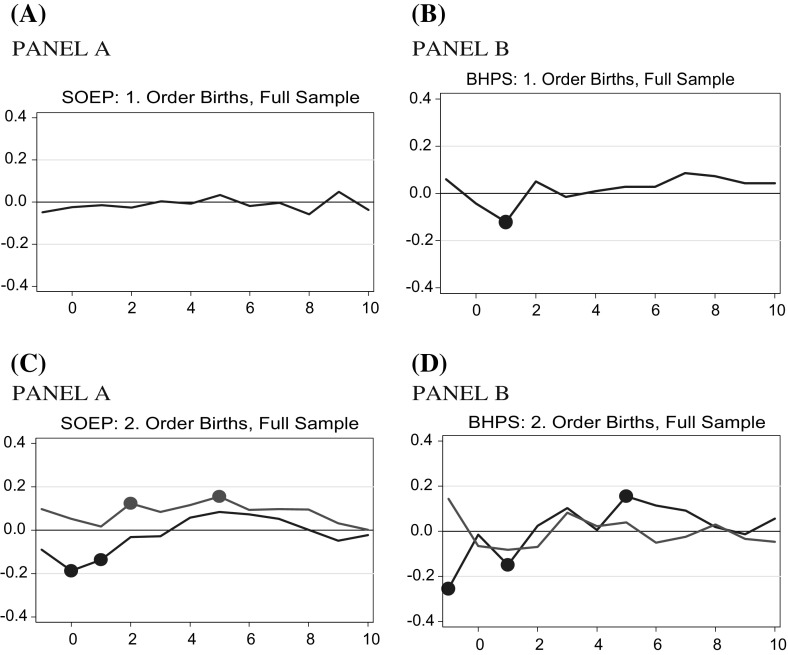
**a**, **b** Happiness and sex of the children. *X*-axis: years before and after a first birth. *Y*-axis: Happiness (scale 0–10). *Solid black line* Coefficients representing the happiness difference between those whose first child was boy versus girl. The *dots* denote *p* < 0.05. Numerical values of the coefficients and *p* values are given in “Appendix”. **c**, **d** Happiness and sex of the children. *X*-axis: years before and after the birth of the second child. *Y*-axis: Happiness (scale 0–10). *Solid black* and *dotted gray lines*: Coefficients representing the happiness difference between those whose first two children were boys (*solid black*) or girls (*dotted gray*) versus those who had one of each. The *dots* denote *p* < 0.05. Numerical values of the coefficients and *p* values are given in “Appendix”

Second, we examine the effects of children’s sex on parents’ happiness. Figure 2a–d present the results graphically, for those who have one and two children and who may progress to a higher order birth, first in Germany and then in the UK. The lines chart the effects on happiness at each time period, where zero is the year that the parent reports the child and go through ten years after the child is born. Figure 2a shows the effect on happiness of having a boy relative to a girl. There are no differences in Germany in parental happiness given the sex of the first child, and this is evident from no dots denoting significant differences from zero. Figure 2b examines the effect of first child’s sex on the happiness of British parents. There is just one time period where a sex difference can be detected. This is one year after the child is reported. Those reporting boys have slightly lower happiness in this year than reporting girls. But there are no differences found for any other years. Moreover, Figs. 1 and 2 show the results by parental characteristics, and these are equally flat as those obtained with the full sample. Overall these results suggest that there are no large sex preferences.

Figure 2c, d chart the happiness of parents that have two boys (solid black line), and two girls (dotted gray line) relative to those that have mixed-sex children. In Germany (Fig. 2c), those who have two boys have significantly lower happiness than those having one boy and one girl, but only in the year reporting the child and the year after. In contrast, those reporting two girls have slightly higher happiness two and five years after having a child than those with mixed-sex children. In the UK (Fig. 2d), there are no differences in happiness between those with two girls and one boy and one girl. However, those with two boys are slightly less happy than parents of mixed-sex children the year before the child, the year after the child, and then are happier than mixed-sex parents five years after a birth. These differences are not large in magnitude, and only appear in a small number of years.

**Fig. 3:**
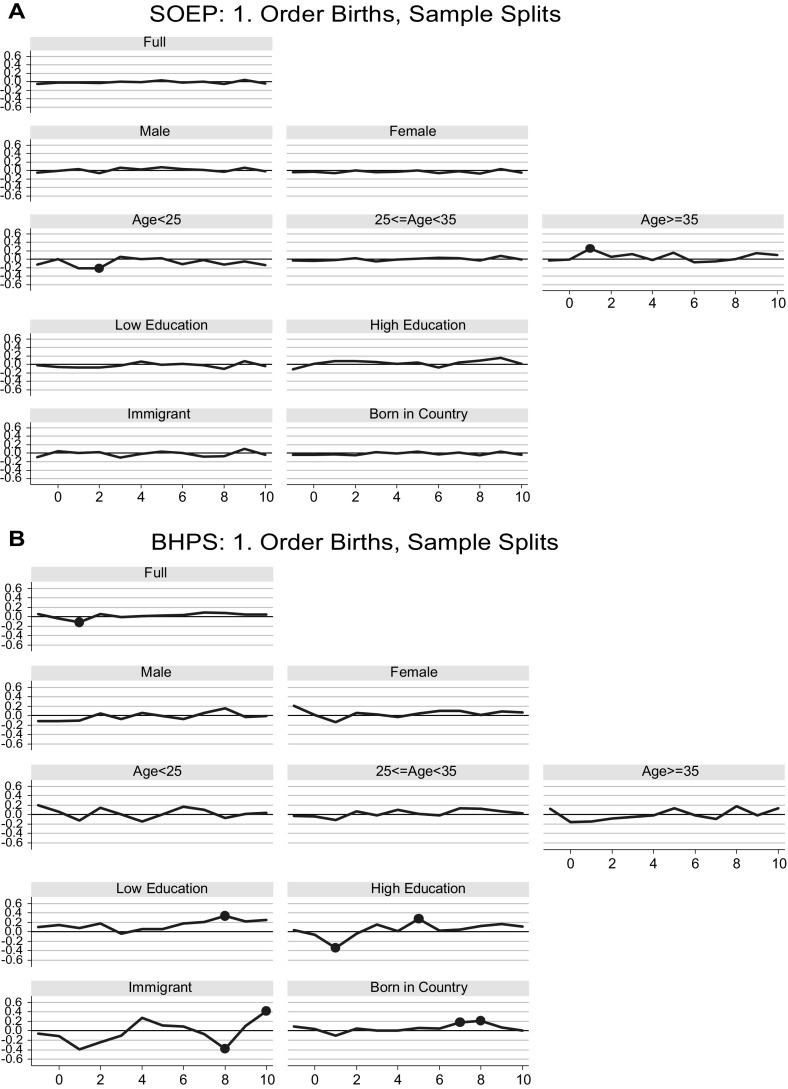
**a** SOEP, **b** BHPS. The *dots* denote *p* < 0.05

**Fig. 4:**
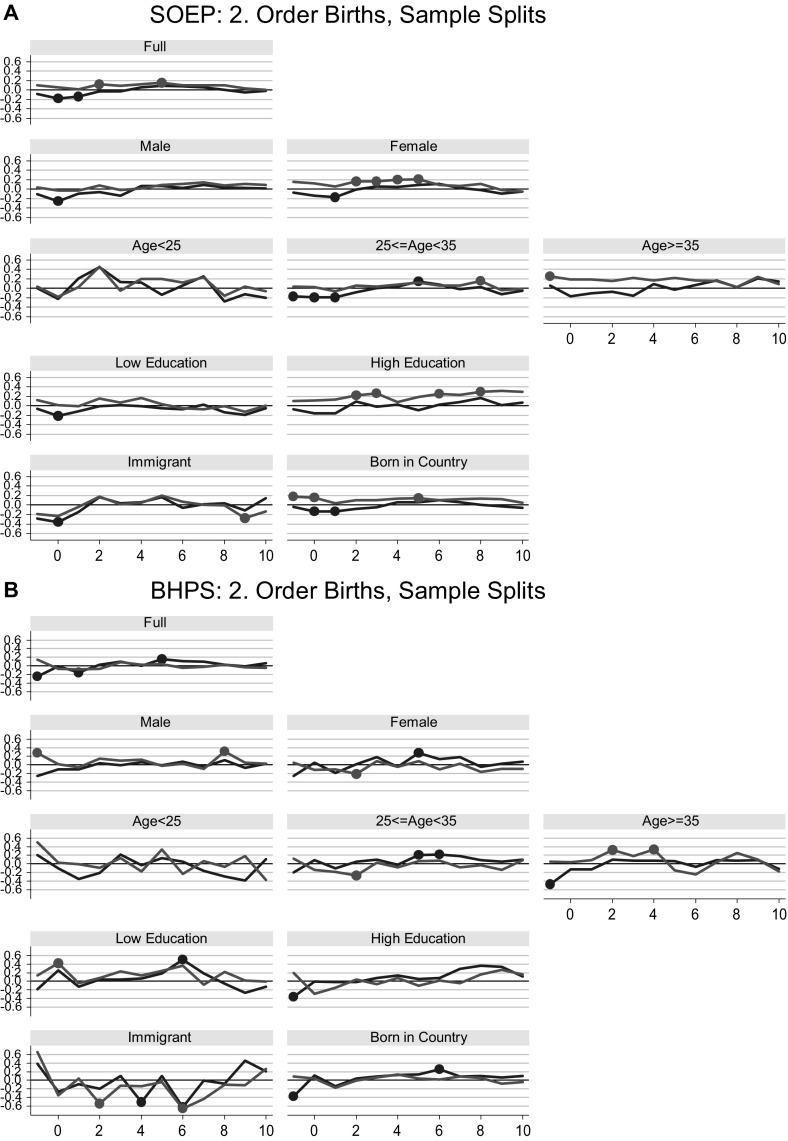
**a** SOEP, **b** BHPS. The *dots* denote *p* < 0.05

We also estimated the effects of children’s sex distribution on happiness by parental characteristics. The results are shown in Figs. 3 and 4 of “Appendix”. We note that in the SOEP, the decrease in happiness associated with two boys is most consistently observed among those aged 25–34 (significant decline in one year before, year of the birth, and year after), and the magnitude of the happiness decline is largest among immigrants. These results are in contrast to the BHPS data, which show no decline in happiness among parents aged 25–34 who had two boys, and among migrants, the decline is observed only on the 4th year after birth. Looking at the effect of two girls on happiness, the results in the SOEP rather suggest slight increases in happiness rather than decreases. However, none of the observed patterns about increased happiness following the birth of a second girl are replicated in the BHPS data.

Overall, the results are largely mixed, and in the handful of cases where the point estimates are significant, the magnitude is not very large. Moreover, the significant effects are in most cases observed only for two boys and not for girls, or vice versa, and in only one of the data sets. This is in sharp contrast to the parity progression results which were consistently in line with prior research and expectations. This leads us to conclude that the differences in happiness generated by variation in the sex distribution of children are quite small. In sum, although we expected to see parents to be less happy with same-sex children, there is little evidence to support this.

## Discussion

Demographers are interested in sex preferences for children because they can skew sex ratios and influence population level fertility, parenting behavior, and family outcomes. In modern European countries, where gender equality is relatively high and parents’ reliance on children in older age has been eroded by public and private pensions, strong preferences for mixed-sex children still emerge in parity progression. Based on parity progression ratios, in most European countries, there are no sex preferences for a first child, but a strong preference for mixed-sex children.

We hypothesized that mixed-sex preferences also influence parental happiness. The topic is important as parents’ disappointment with a second child of the same sex as the first could have negative effects for parents and children. Testing this question with large panel data sets in Germany and the UK, we find no differences in parental happiness given the sex of the first child, and minimal differences after a second child that only appear in a very small number of years. We also tested whether the results are stronger among subpopulations of parents (age, education, sex, and nativity). For this analysis, the results were mixed and of very small magnitude. While mixed-sex preferences influence fertility (likelihood of having a third birth), they have little impact on parental happiness.

The fact that there are no differences in parental happiness by the sex of a first child maps onto the fact that there are no differences in parity progression to a second child. However, there is a divergence between results for parental happiness and parity progression after a second birth. It is possible that even strong sex preferences do not translate into differences in parental happiness due to countervailing forces as two same-sex children may also positively influence parental happiness. On the one hand, parents may be disappointed to have a second child of the same sex as the first. But on the other hand, this disappointment may be short-lived as has been found for many other life events (Clark et al. 2008). It could also be that positive factors emerge regarding having same-sex children such as the ability to reuse gendered children’s items or participate in similar gendered activities. Another potential explanation for the disparity between the small effect of sex composition on happiness, but the large effect on subsequent childbearingis that parents perceive that they will benefit in the long run from having at least one child of each sex. Parents’ thoughts about long-run happiness and the sex composition of children can be tested with other data, but was not able to be tested in our study.

We hypothesized that two children of the same sex would decrease parental happiness, as parity progression ratios after two children suggest a disappointment in the sex of the second child. We consider that null finding—little if any effect on parental happiness—as positive. A predicted (but not observed) decline in parental happiness would have obviously indicated a decline in well-being of the parent, and indirectly perhaps also a decline in the well-being of the child or both children, as parental well-being is an important determinant of child development. A lack of such a decline in parental well-being is a welcome null finding, as it suggests that parents are happy with the children they have, regardless of their sex.

## Appendix

**Table 3:** Numerical values of the coefficients and *p* values for Fig. 2a, b, first order births

Lag	SOEP		BHPS	
	*B* boy	*p* value	*B* boy	*p* value
−1	−0.048	0.314	0.060	0.460
0	−0.023	0.589	−0.042	0.466
1	−0.015	0.732	−0.120	0.046
2	−0.026	0.566	0.051	0.378
3	0.005	0.911	−0.014	0.799
4	−0.006	0.885	0.010	0.864
5	0.033	0.456	0.028	0.615
6	−0.019	0.667	0.029	0.584
7	−0.002	0.956	0.085	0.123
8	−0.057	0.184	0.073	0.190
9	0.050	0.248	0.043	0.446
10	−0.037	0.398	0.043	0.446

**Table 4:** Numerical values of the coefficients and *p* values for Fig. 2c, d, second order births

Lag	SOEP				BHPS			
	*B* 2 boys	*p* value	*B* 2 girls	*p* value	*B* 2 boys	*p* value	*B* 2 girls	*p* value
−1	−0.088	0.168	0.098	0.139	−0.253	0.007	0.145	0.134
0	−0.187	0.002	0.053	0.385	−0.014	0.846	−0.064	0.379
1	−0.136	0.025	0.018	0.774	−0.150	0.049	−0.082	0.305
2	−0.032	0.602	0.124	0.044	0.026	0.730	−0.069	0.374
3	−0.027	0.657	0.084	0.171	0.103	0.161	0.084	0.283
4	0.058	0.343	0.117	0.064	0.005	0.946	0.023	0.774
5	0.084	0.169	0.155	0.013	0.156	0.033	0.040	0.595
6	0.073	0.217	0.095	0.119	0.114	0.130	−0.051	0.518
7	0.053	0.380	0.098	0.116	0.092	0.224	−0.024	0.763
8	0.002	0.969	0.097	0.121	0.020	0.794	0.031	0.695
9	−0.048	0.444	0.032	0.617	−0.013	0.853	−0.033	0.665
10	−0.022	0.716	0.003	0.963	0.057	0.447	−0.047	0.553

Figures 3 and 4 show the results from interaction models that estimate whether the impact of children’s sex distribution on happiness depends on individual characteristics. The *X*- and *Y*-axes and color coding are the same as for Fig. 2a–d.
